# Better Safe Than Sorry? Exploratory Associations Between Fear-Learning Processes and Later Paranoia in Threat-Exposed Adolescents in Standardized Virtual Reality Environments

**DOI:** 10.1093/schizbullopen/sgag036

**Published:** 2026-09-12

**Authors:** Cansel Inan, Celine Samaey, Kristof Vansteelandt, Aleksandra Lecei, Charlotte Gayer-Anderson, Lucia Valmaggia, Ruud van Winkel

**Affiliations:** Center for Clinical Psychiatry, Research Group Psychiatry, Department of Neuroscience, KU Leuven, ON5, Herestraat 49, box 1029, Leuven 3000, Belgium; Center for Clinical Psychiatry, Research Group Psychiatry, Department of Neuroscience, KU Leuven, ON5, Herestraat 49, box 1029, Leuven 3000, Belgium; Laboratory for Biological Psychology, Department of Brain and Cognition, KU Leuven, Tiensestraat 102 - box 3714, Leuven 3000, Belgium; Research Group Psychiatry, Department of Neurosciences, KU Leuven, ON5, Herestraat 49, box 1029, Leuven 3000, Belgium; Center for Clinical Psychiatry, Research Group Psychiatry, Department of Neuroscience, KU Leuven, ON5, Herestraat 49, box 1029, Leuven 3000, Belgium; Department of Health Services and Population Research, Institute of Psychiatry, Psychology & Neuroscience, King’s College London, 16 De Crespigny Park, London SE5 8AF, United Kingdom; ESRC Centre for Society and Mental Health, King’s College London, Strand, London, WC2R 2LS, United Kingdom; Orygen, The National Centre of Excellence in Youth Mental Health, Parkville, Victoria, 35 Poplar Road, Parkville VIC 3052, Australia; Centre for Youth Mental Health, The University of Melbourne, 35 Poplar Road, Parkville VIC 3052, Australia; Department of Psychology, Institute of Psychiatry, Psychology & Neuroscience, King’s College London, 15 De Crespigny Park, London SE5 8AF, United Kingdom; Center for Clinical Psychiatry, Research Group Psychiatry, Department of Neuroscience, KU Leuven, ON5, Herestraat 49, box 1029, Leuven 3000, Belgium

**Keywords:** state paranoia, fear learning, childhood adversity, prospective longitudinal study

## Abstract

**Background:**

Childhood threat exposure (CTE) has been linked to altered fear-learning, yet its prospective role in adolescent paranoia remains unclear. This study examined whether threat–safety discrimination (TSD) and fear generalization (FG) predict virtual reality (VR)-assessed state paranoia 18 months later, and whether CTE and gender moderate these associations.

**Methods:**

A total of 120 adolescents (aged 12-16) participated at wave 1, completing a classical conditioning and FG task and reporting CTE. At wave 2, 97 participants completed a VR social environment assessing state paranoia. Linear regression models tested TSD and FG main effects, followed by moderation by CTE, and the FG × CTE × gender interactions.

**Results:**

Neither TSD (β = 0.14, *P* = .198) nor FG (β = −0.07, *P* = .530) predicted wave 2 paranoia at group level. However, CTE significantly moderated the association between TSD and later paranoia (β = 0.21, *P* = .035), whereby adolescents with higher CTE and better discrimination showed higher paranoia. A significant FG × CTE × gender interaction (β = 0.53, *P* = .044) revealed divergent pathways: in boys, higher FG predicted lower paranoia (*F*(1, 84) = 7.34, *P* = .008), particularly with higher CTE, which was absent in girls.

**Conclusion:**

Exploratory interactions, partly opposite to our hypotheses, suggested that better TSD and reduced FG were associated with higher paranoia in threat-exposed youth, particularly boys. This pattern may reflect a “better safe than sorry” strategy involving fine-grained appraisal of threat and safety cues, potentially adaptive in unpredictable and threatening environments but at the cost of greater distrust.

## Introduction

Childhood adversity (CA) encompasses a broad range of harmful experiences during development, including threat-related events such as abuse, violence, and victimization, as well as deprivation-related experiences like neglect and lack of cognitive or emotional stimulation.^1–3^ Importantly, the dimensions of threat and deprivation are thought to affect neurodevelopment through different pathways, with threat primarily altering fear and threat-processing circuits, and deprivation impacting cognitive and emotional regulatory systems.^1^^,^^2^^,^^4^ This distinction is critical, as emerging evidence suggests that threat exposure has a particularly strong influence on the development of altered threat–safety discrimination (TSD), the ability to distinguish dangerous from safe environmental cues, and overgeneralization of fear-associated events (fear generalization [FG]).^5–8^

These alterations may be particularly pronounced in social contexts, as evidenced by impaired neural differentiation between threatening and neutral social cues, emphasizing the targeted impact of CA on social fear processing.^6^^,^^7^ Altered threat processing, linked to neural changes in the hippocampus and amygdala^9^ may thus increase risk for psychopathology, such as post-traumatic stress disorder, social anxiety, or psychosis.^10–13^

While the effects of CA on threat processing have been well documented in adults,^1^^,^^6–8^ less is known about how these mechanisms operate during adolescence, a critical developmental period marked by ongoing neurobiological and emotional maturation when maladaptive fear responses may solidify, influencing long-term mental health trajectories.^14^^,^^15^ Adolescence presents a unique window to study how childhood threat exposure (CTE) impacts fear learning, generalization and distinction before the onset of psychopathology, enabling identification of early risk and resilience factors.

Within social contexts, altered threat processing may manifest as paranoia, characterized by heightened social hypervigilance and beliefs that others intend harm.^16–18^ From an environmental calibration perspective, paranoia may represent an adaptively shaped response to unpredictable or threatening environments following CA.^19–21^ Disruptions in TSD and heightened FG may contribute to this process by increasing the tendency to interpret ambiguous social cues as threatening, thereby reinforcing paranoid ideation. Although impaired TSD and heightened FG have been extensively linked to post-traumatic stress disorder and anxiety disorders^10^^,^^22–25^ research directly examining their prospective association with paranoia remains scarce. To our knowledge, no longitudinal study has investigated whether individual differences in fear-learning processes following CTE shape trajectories of paranoia during adolescence, leaving a critical gap in understanding the developmental pathways from altered threat processing to socially expressed psychopathology.

To address these gaps in the literature, the EMBRACE study was set up as a prospective investigation of adolescents aged 12-16 years, examining how CA influences TSD and FG, 2 key processes underlying adaptive fear learning and the development of psychopathology. In the context of this study, TSD refers to the ability to distinguish between stimuli that signal potential danger (threat) and those that signal safety. It can be assessed by pairing a conditioned stimulus (CS+) with an aversive stimulus (eg, electrical shock) and ensuring that another stimulus (CS−) signals safety by never being paired with the shock. Fear generalization refers to the tendency for an individual to extend conditioned fear responses from the original threat (CS+) to similar, yet nonthreatening stimuli (generalization stimuli [GSs]). In a typical fear learning scenario, this generalization is expected to occur only within a narrow range of stimuli that are highly similar to the CS+. Wave 1 data from the EMBRACE study revealed that adolescents with greater CA exposure exhibited a reduced ability to distinguish threat from safety cues, resulting in overgeneralized fear responses to stimuli similar but not identical to original threats, the latter of which were different in boys and girls.^5^ These changes reflected alterations in fear learning and emotional regulation rather than perceptual limitations. Notably, boys exposed to adversity tended to generalize fear more toward safety cues, suggesting difficulty recognizing safety, whereas girls showed greater generalization toward threat cues, reflecting heightened sensitivity to danger. These sex-specific patterns may underlie the increased vulnerability to anxiety and paranoia observed particularly in adolescent girls following CA.^5^

Building upon this study, a longitudinal wave 2 of the study sample of 120 adolescents aged 12-16 utilized virtual reality (VR) to assess state paranoia across 2 distinct social environments, a school canteen (at wave 1) and a bar (wave 2). Virtual reality provided a unique and ecologically valid platform to simulate social interactions and assess real-time paranoid responses in controlled yet realistic settings in that context.^26–32^ Findings indicated that wave 1 state paranoia strongly predicted wave 2 state paranoia, highlighting the stability and predictability of paranoid ideation over time despite different social contexts.^26^ Moreover, CTE moderated the association between state paranoia and anxiety over time: adolescents with higher wave 1 paranoia and greater CTE experienced less anxiety at wave 2, suggesting distinct developmental trajectories of paranoia and anxiety influenced by early adversity.^26^ These results emphasize that paranoia is rooted in cognitive processes tied to social environmental perception and may function adaptively in high-threat contexts with higher levels of paranoia.^26^ Specifically, higher attention to threat signals, particularly under conditions of higher threat exposure, might exacerbate paranoia by reinforcing maladaptive cognitive biases, leading adolescents to perceive ambiguous social cues as threatening.^26^^,^^33^

Together, these complementary studies illustrate how early CTE disrupt fear-learning processes and shapes paranoia development across adolescence. However, no study has yet prospectively examined whether individual differences in TSD and FG after CTE predict later state paranoia in ecologically valid social environments, nor whether these associations differ by gender.

The present prospective study, therefore, aimed to test whether fear-learning processes assessed in mid-adolescence forecast state paranoia 18 months later in VR social contexts. Drawing on prior work showing that CA is associated with impaired TSD and heightened FG,^5^ that paranoia may represent an adaptively calibrated response to threatening environments,^19^^,^^20^^,^^26^ and that there are gender-specific patterns in the impact of adversity on FG,^5^ we formulated the following hypotheses: (1) Main effects of fear learning on later state paranoia. Adolescents with poorer TSD and greater FG at wave 1 would show higher levels of state paranoia at wave 2. This follows from evidence that reduced TSD and broadened FG are linked to overestimation of threat and internalizing psychopathology. (2) Moderation by CTE. The prospective associations between fear-learning indices (TSD and FG) and wave 2 state paranoia would be stronger in adolescents with higher levels of CTE. This is consistent with environmental calibration accounts, which propose that prior threat sensitizes threat-processing systems and increases the impact of fear-learning biases on later appraisals. (3) Gender-specific effects for FG. Based on earlier EMBRACE findings of sex differences in FG after CA,^5^ we expected that higher FG would be more strongly associated with state paranoia in girls than in boys, particularly at higher levels of CTE. (4) Prediction of change in state paranoia over time. Finally, we hypothesized that, even after controlling for baseline state paranoia, fear-learning indices, especially FG in the context of higher CTE, would predict an increase in state paranoia across the 18-month follow-up, indicating that these processes contribute to the persistence and amplification of paranoid ideation during adolescence.

## Methods

### Sample

Participants were recruited from the general population, schools, and specialized support centers (nonclinical organizations offering assistance in cases of suspected abuse or victimization, such as Vertrouwenscentra Kindermishandeling). Exclusion criteria encompassed medical or neurological disorders, claustrophobia, intellectual disability, pregnancy, use of psychotropic medication, acute suicidality, and ongoing abuse. Inclusion criteria required fluency in Dutch and being within the target age range (12-16). Most participants came from the general population, with 9 participants recruited from a specialized center. Therefore, the sample represents a general population group that includes adolescents exposed to greater adversity. Assent was obtained from the participants, and informed consent was obtained from their parents or guardians. They received compensation of €10 per hour, and travel costs were reimbursed. The study was approved by the UZ/KU Leuven Medical Ethics Committee (S62124). Data collection took place in 2 waves: the first from August 2020 to July 2022, and the second from June 2022 to January 2024.

### Study Design

This study used a prospective longitudinal design consisting of 2 assessment waves spaced approximately 18 months apart. During the first wave, TSD and FG were evaluated, and CA was measured using a validated semi-structured interview. In the second wave, state paranoia was assessed immediately following participants’ completion of the VR tasks, while they were still immersed in the virtual environment.

### Fear Conditioning Paradigm

Fear conditioning and generalization were assessed using a classical conditioning paradigm adapted from Lissek et al.^11^ In this task, participants were presented with 10 circles varying in size, ranging from 2 to 4.7 inches. Across participants, either the third or the eighth circle was designated as the conditioned stimulus (CS+), with the other serving as the safety stimulus (CS−), counterbalanced between participants. These positions were chosen to avoid the use of extreme stimuli, thereby minimizing potential perceptual or expectancy biases that could arise if the CS+ or CS− were located at the ends of the stimulus continuum.^5^^,^^11^ A mild electrical shock, applied to the nondominant wrist, served as the unconditioned stimulus (US). Before the experiment, participants individually calibrated the shock intensity to a level that was “uncomfortable but not painful.”

The paradigm was structured into 3 phases: habituation, acquisition, and generalization, assessing fear learning both behaviorally and physiologically. In each trial, participants rated their expectation of receiving a shock on a 10-point Likert scale (1 = Definitely no shock, 10 = Definitely a shock). A 40-ms, 95-dB startle probe was presented in half of the trials and a quarter of the intertrial intervals to evoke a fear-potentiated startle. Due to technical difficulties during psychophysiological recording, the fear-potentiated startle responses could not be accurately interpreted and were thus excluded from the analysis. Therefore, analyses relied on behavioral expectancy ratings.

In the *habituation* phase, the CS+ and CS− were shown 4 times each, with no shock. Each trial began with a 1-s fixation cross, followed by the CS displayed for 8 s. After 2 s, a rating scale appeared, and participants indicated their shock expectancy. The interstimulus interval ranged between 2.2 and 3.2 s. All trials were semi-randomized with no more than 2 identical trials in a row.

During the *acquisition* phase, the CS+ was paired with a 100 ms electrical shock in 75% of trials, with CS+ and CS− shown 8 times each. The shock co-terminated with the CS+ stimulus (Figure 1).

**Figure 1:**
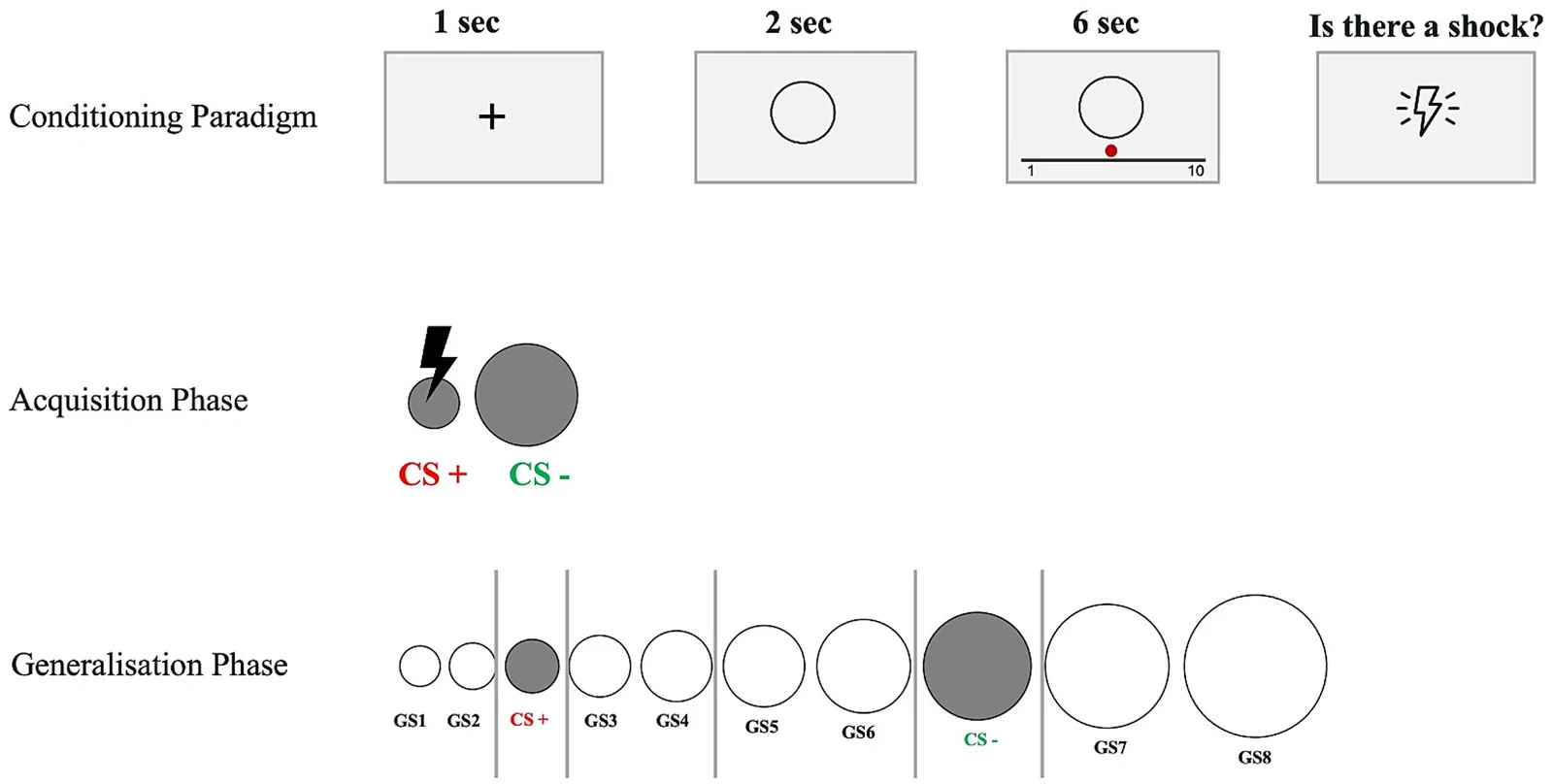
*Conditioning Paradigm* Used in the Fear-Learning Task

In the *generalization* phase, both CS+ and CS− were shown again with 50% reinforcement for the CS+. Additionally, 8 GSs were shown 4 times each. The GSs and CS− were never paired with the shock. Trials were semi-randomized and grouped into 4 blocks of 12 trials (2 CS+, 2 CS−, and 1 of each GS) (Figure 1). The task was programmed in Python using PsychoPy.^34^

After the generalization phase, participants rated all CSs and GSs on US expectancy, fear, valence, and arousal, using a 10-point Likert scale for the first 2 and the self-assessment manikin for the latter 2.^36^

A fixation cross appeared at the center of the screen for 1 s, after which a stimulus (a circle) was displayed for 8 s. Two seconds after the stimulus appeared, a rating scale was shown beneath it, asking participants to indicate how likely they thought an electric shock would occur. The scale disappeared once a response was given or, if no response was made, when the stimulus ended. During the *acquisition phase*, only the danger and safety cues were shown, whereas during the *generalization phase*, all stimuli were presented. The assignment of stimuli was counterbalanced across participants. Adapted from Qiao et al.^7^ and Samaey et al.^5^

### Virtual Reality Environments

At wave 1, participants engaged in social interactions within a VR school canteen, designed by the KCL Institute of Psychiatry, Psychology, and Neuroscience VR Lab and built by Virtualware.^27^ The virtual environment was adapted to reflect the Belgian school context; for instance, avatars wore casual clothing instead of school uniforms, and their ethnic diversity mirrored that typically seen in Belgian schools. Participants used a gaming joystick to interact with virtual peer avatars in both individual and group settings. They encountered neutral yet ambiguous situations, such as peers either gazing at them or laughing among themselves. Participants were instructed to form impressions of the avatars and reflect on the potential thoughts or intentions of these virtual peers. The task began with a bell signaling the start of the break, followed by a brief navigation through a corridor to help participants familiarize themselves with the virtual space and controls. They then followed a series of voice-guided instructions, leading them through various neutral or ambiguous social scenarios, such as being approached by a peer with a question, being informed a seat was occupied, and approaching a group of students talking together. The session concluded with another bell, signaling the end of the break and the return to class. After leaving the canteen, participants answered a series of questions assessing their subjective experiences during the task. Further details on the paradigm can be found in Gayer-Anderson et al.^27^

During the wave 2 session, conducted approximately 18 months after the initial assessment, participants’ social interactions and paranoid experiences were examined in a VR bar environment, also created by the KCL Institute of Psychiatry, Psychology, and Neuroscience VR Lab and built by Virtualware.^35^ The virtual bar depicted a moderately crowded evening setting, populated by young adult avatars of diverse ethnic backgrounds dressed in casual social attire. Avatars were programmed to display neutral yet socially ambiguous behaviors, such as briefly glancing toward the participant, engaging in conversations with one another, smiling or laughing within groups, or standing alone while observing the room. No avatar engaged in explicitly hostile or overtly threatening behavior.

The task began outside the bar entrance, allowing participants to familiarize themselves with the virtual surroundings and navigation controls before entering the social setting. Participants then entered the bar and were free to move around the environment using a joystick, encountering avatars individually and in small groups. As in wave 1, they were instructed to observe the social environment, form impressions of the avatars, and consider what others in the bar might be thinking about them. The scenarios were designed to remain socially ambiguous, thereby eliciting individual differences in threat interpretation and paranoid ideation. Prior research using this VR bar has shown that environmental factors such as crowding and the hostility of avatars can heighten paranoid ideation, providing insights into social perception and mental health.^28^^,^^32^^,^^35^^,^^37^ The VR environments were presented in color through a lightweight headset, offering a 1280 × 1024 resolution, 360° view, and a 60 Hz refresh rate.

### Measures

#### Threat–Safety Discrimination (TSD)

For the TSD measurement, the *acquisition* phase was analyzed. The data were scored as follows: Threat Discrimination Index (TDI) = Mean (US Expectancy for CS+) − Mean (US Expectancy for CS−), where a higher value reflects better TSD. This method was adapted from Samaey et al. (2025), who used a similar approach to assess TSD in adolescents exposed to CA.

#### Fear Generalization (FG)

To quantify individual differences in FG, based on the *generalization* phase, we computed a generalization index (GI) designed to capture the magnitude of conditioned responding to GSs relative to the conditioned stimulus (CS+). Specifically, we focused on the middle portion of the generalization gradient, GS3-GS6, which typically exhibits linear properties in perceptual similarity studies.^10^^,^^38^^,^^39^ For each participant, we calculated the average difference between the mean response to each of these GSs and the participant’s mean response to the CS+, resulting in the following formula:


$$ \frac{\sum_{i=3}^6\overline{\Big({\mathrm{GS}}_i}-\overline{\mathrm{CS}+}\Big)}{4} $$

This index offers several advantages over previously proposed methods.^10^^,^^38^^,^^39^ First, it is centered on individual CS+ reactivity, controlling for wave 1 differences in response magnitude across participants, a critical feature when examining between-subject associations, such as with paranoia. Second, it avoids the distortions introduced by ratio-based scaling (eg, dividing by CS+).

We chose this method over other indices for both conceptual and statistical reasons. While Lenaert et al.^33^ proposed a generalization “mass” index that aggregates across all GSs, their approach does not account for gradient shape or regional specificity and includes outer GSs that may be less reliable. Similarly, Kaczkurkin and Lissek’s^38^ deviation-from-linearity index and the group-level gradient analyses used in Lissek et al.^10^^,^^11^ are either ambiguous in interpretation (eg, GI = 0 may reflect both linear and U-shaped gradients), require a CS−, or are not well-suited for individual-level statistical modeling. In contrast, our GI provides a theoretically grounded and flexible measure of generalization magnitude that retains meaningful interindividual variability and is therefore well-suited for exploring its association with downstream cognitive-affective traits such as paranoia.

#### State Paranoia in the VR Environment

State paranoia was assessed using the State Social Paranoia Scale (SSPS) at the end of each VR task.^29^ It includes 5 neutral, 5 positive, and 10 persecutory (paranoid) thoughts, and perceptions of participants in the social environment, rated on a 5-point scale. In the wave 1 study, 5 persecutory thoughts were assessed to minimize the burden for young adolescents^27^ (Table S1). At wave 2, the full 10 persecutory items were used by Freeman et al.^29^ The sum of the persecutory items was analyzed.

#### Childhood Adversity (CA)

Childhood adversity was assessed using an adapted version of the Juvenile Victimization Questionnaire—2nd Revision (JVQ-R2),^40^ which covers 34 types of youth victimization, including crime, maltreatment, peer and sibling victimization, sexual victimization, and witnessing violence. Participants answered screener questions with “yes” or “no,” followed by questions on the age of onset, frequency, and duration of adverse events, as well as their past and current effects. Frequency was rated on a 5-point scale, and a composite score was created based on the amount and frequency of adversities.^5^^,^^41^ A score of 0 was given for any adversity not experienced. The mean frequency of adverse events for each category was calculated and summed, with higher scores indicating greater exposure to CA. Since our hypothesis focuses specifically on CTE, the childhood threat frequency subscale was used in all analyses. Thus, the childhood threat frequency, which we called CTE, was used at wave 1.

### Internal Consistency of Measures

Reliability analyses were conducted for all self-report instruments used in this study. The shortened 5-item version of the SSPS administered at wave 1 showed good internal consistency (α = 0.84, 95% CI [0.78, 0.88]), whereas the full 10-item version used at wave 2 demonstrated excellent internal consistency (α = 0.92, 95% CI [0.90, 0.94]). The complete SSPS exhibits high internal consistency (α = 0.91), strong temporal stability (test–retest *r* = 0.73), and good convergent validity based on both interviewer assessments and participants’ self-reports.^29^ A post hoc comparison of SSPS mean scores across waves revealed no significant difference in state paranoia levels between wave 1 (*M* = 1.91, SD = 0.68) and wave 2 (*M* = 1.92, SD = 0.85), *t*(89) = 0.75, *P* = .45, 95% CI [−0.11, 0.24]. For comparability across waves, mean item scores were used, given that the wave 1 assessment included 5 persecutory items, whereas the wave 2 assessment included the full 10-item scale.

### Statistical Analysis

All analyses were conducted in RStudio (version 2024.12.1 + 563). The primary R packages used for data manipulation included tidyr (1.3.1), tidyverse (2.0.0), and dplyr (1.1.4). For the analysis, lm.beta (1.7-2), dplyr (1.1.4), and knitr (1.45) were utilized, while data visualization was done using ggplot2 (3.5.1).

## Prospective Associations: Predicting Wave 2 State Paranoia From Wave 1 Measures

### Threat–Safety Discrimination

(1) Linear regression analyses evaluated the prospective association between wave 1 TSD and wave 2 state paranoia. Initial analyses examined the direct relationship, followed by (2) a multiple regression with the moderation by wave 1 CTE. Gender and age were kept as covariates.

### Fear Generalization

(3) Similarly, linear regression analyses were conducted to assess the prospective association between wave 1 FG and wave 2 state paranoia. Initial evaluations were followed by (4) a multiple regression moderation analysis involving wave 1 CTE and then (5) a 3-way interaction between wave 1 FG × CTE × gender. The use of a 3-way interaction was based on the findings of Samaey et al.^5^, who observed gender differences in the way that CA influenced FG, with boys and girls showing distinct patterns of fear responses. Age was kept as a covariate.

## Change in Symptoms: Examining Changes in State Paranoia Over Time While Controlling Wave 1 State Paranoia

### Threat–Safety Discrimination

(1) Linear regression analyses examined whether TSD predicted increase in state paranoia symptoms over time, *controlling for wave 1 state paranoia*. (2) Multiple regression moderation by wave 1 CTE was also evaluated. Gender and age were kept as covariates.

### Fear Generalization

(3) Furthermore, linear regression analyses investigated FG as a predictor of an increase in state paranoia symptoms over time, again *controlling for wave 1 state paranoia*. (4) Multiple regression moderation by wave 1 CTE and then (5) a 3-way interaction between wave 1 FG × CTE × gender was also assessed. Age was kept as a covariate.

## Results

The study included 120 adolescents between 12 and 16 years old. A total of 97 participants (41 males and 56 females), with an average age of 15.44 years (SD = 1.36) at wave 2, completed both assessment waves (see Table 1).

**Table 1:** Descriptive Statistics for Sample Demographics in Both Waves

**Demographics**	**Subcategory**	**Wave 1**				**Wave 2**			
		***n***	***%***	***Mean***	***SD***	***n***	***%***	***Mean***	***SD***
Total *n*		120				97			
Age				13.93	1.36			15.44	1.36
Gender	Male	53	44.17			41	42.27		
	Female	66	55			56	57.73		
	Other	1	0.83						
Birthplace	Belgium	109	90.83			88	90.72		
	Western/Northern Europe	2	1.67			1	1.03		
	Eastern Europe	3	2.5			2	2.06		
	Other	6	5			6	6.19		
Ethnicity	White	109	90.83			87	89.69		
	Middle Eastern/Arab	3	2.5			3	3.09		
	Asian	2	1.67			2	2.06		
	Mixed Ethnic Groups	4	3.33			3	3.09		
	Unknown/Prefer not to say	2	1.67			2	2.06		
State Paranoia		115		9.51	3.42	94		19.2	8.49
CTE		120		1.02	1.69	96			

Abbreviation: CTE, childhood threat exposure.

Participants reported experiencing a moderate degree of immersion during the VR task (*M* = 3.47 in wave 1) and indicated average satisfaction with the VR social interaction experience (*M* = 3.31 in wave 1 and *M* = 3.25 in wave 2).

Results from the hierarchical regression models are presented sequentially, beginning with main effects and 2-way interactions, followed by the inclusion of the 3-way interaction term.

## Prospective Associations Between Wave 1 Measures and Wave 2 State Paranoia

### Threat–Safety Discrimination

In the main effect model, wave 1 TSD was not significantly associated with wave 2 state paranoia (β = 0.14, SE = 0.11, 95% CI: [−0.07, 0.35], *P* = .198), controlling for age and gender.

However, in the second model, a significant interaction emerged between wave 1 TSD and wave 1 CTE (β = 0.21, SE = 0.10, 95% CI: [0.02, 0.40], *P* = .035), indicating that the association between TSD and later state paranoia depended on the level of CTE: those with greater exposure and better TSD displayed higher levels of state paranoia at wave 2 (Figure 2).

**Figure 2:**
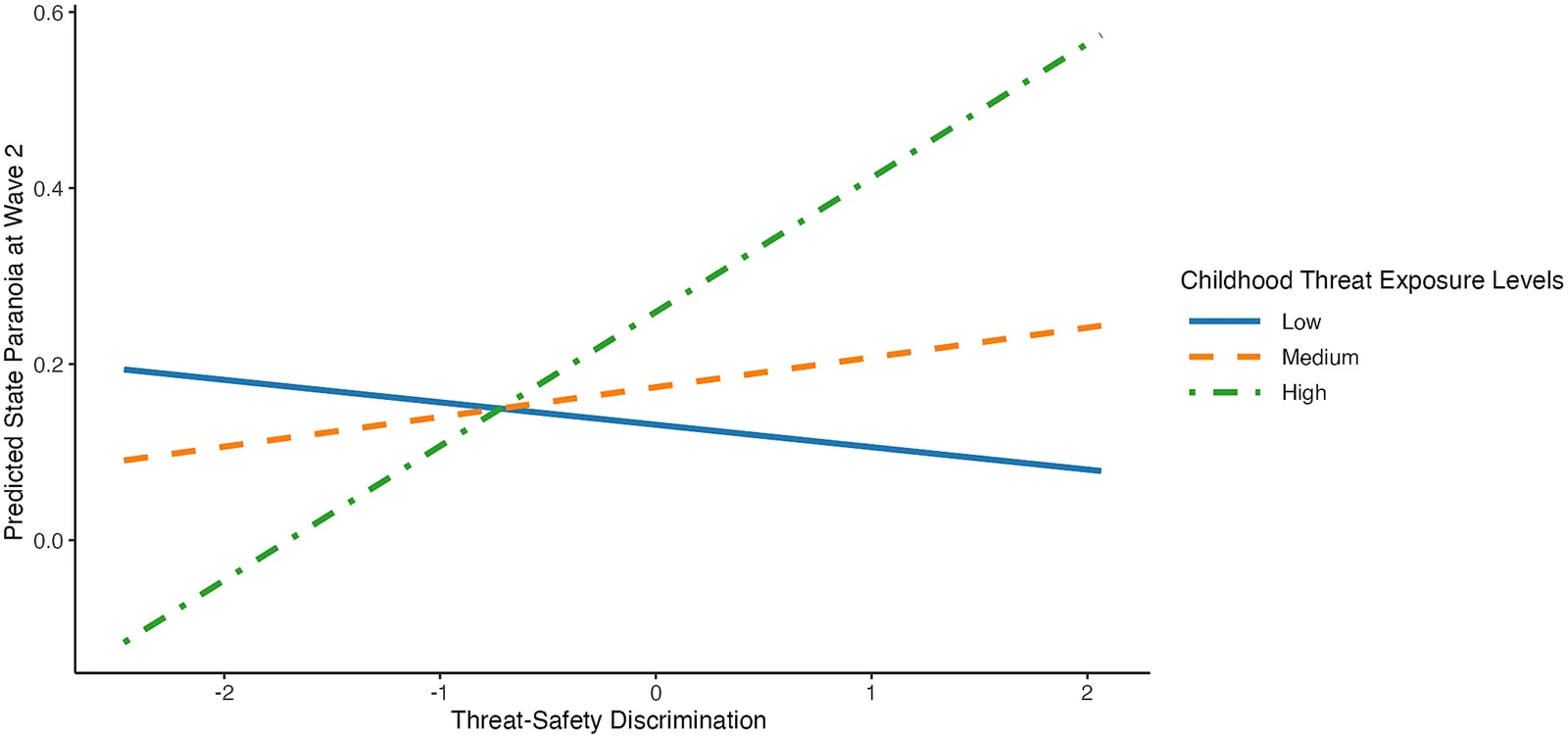
Interaction Effect Between Threat–Safety Discrimination and Childhood Threat Exposure on Wave 2 State Paranoia. *Note*. CTE was modeled as a continuous variable. For illustrative purposes, simple slopes are plotted at the 25th (low), 50th (medium), and 75th (high) percentiles of its distribution. Abbreviation: CTE = childhood threat exposure.

### Fear Generalization

In the initial model including main effects, wave 1 FG was not significantly associated with wave 2 state paranoia (β = −0.07, SE = 0.11, 95% CI [−0.29, 0.15], *P* = .530). Similarly, neither gender nor age emerged as a significant predictor.

The second model, which consisted of the 2-way interaction between wave 1 FG and wave 1 CTE, was also non-significant (β = −0.17, SE = 0.12, 95% CI [−0.41, 0.07], *P* = .157), indicating no clear moderation effect.

However, similar to Samaey et al.^5^, the full regression model including 3-way interaction between wave 1 FG, wave 1 CTE, and gender was statistically significant (β = 0.53, SE = 0.26, 95% CI [0.02, 1.05], *P* = .044). This indicated that the association between FG and later state paranoia varies as a function of both gender and CTE.

The FG × CTE × gender interaction explained an additional 4.3% of the variance (Δ*R*^2^ = .043; Cohen’s *f*^2^ = .05). The estimated interaction was modest in magnitude (β = 0.53, 95% CI [0.02, 1.05], *P* = .044), with a relatively wide confidence interval indicating uncertainty regarding the size of the effect.

To interpret this interaction, post hoc tests were conducted using the full model (Table 2). Joint tests of the conditional effects showed that the FG × CTE interaction at wave 1 was significant in boys, *F*(1, 84) = 7.34, *P* = .008, but not in girls, *F*(1, 84) = 0.02, *P* = .882. Similarly, the simple effect of wave 1 FG was significant in boys, *F*(1, 84) = 6.96, *P* = .010, but not in girls, *F*(1, 84) = 1.31, *P* = .256.

**Table 2:** Post Hoc Tests of Conditional Effects Within Gender

**Gender**	**Effect**	***F*(1, 84)**	***P***
**Boys**	FG	6.96	**.010**
	FG × CTE	7.34	**.008**
**Girls**	FG	1.31	.256
	FG × CTE	0.02	.882

Abbreviations: CTE, childhood threat exposure; FG, fear generalization.

As shown in Figure 3, in boys, the association between FG and state paranoia was significantly negative at high levels of CTE, whereas this association was weak or absent at low levels of CTE. In girls, the association between FG and state paranoia remained weak and non-significant across levels of CTE.

**Figure 3:**
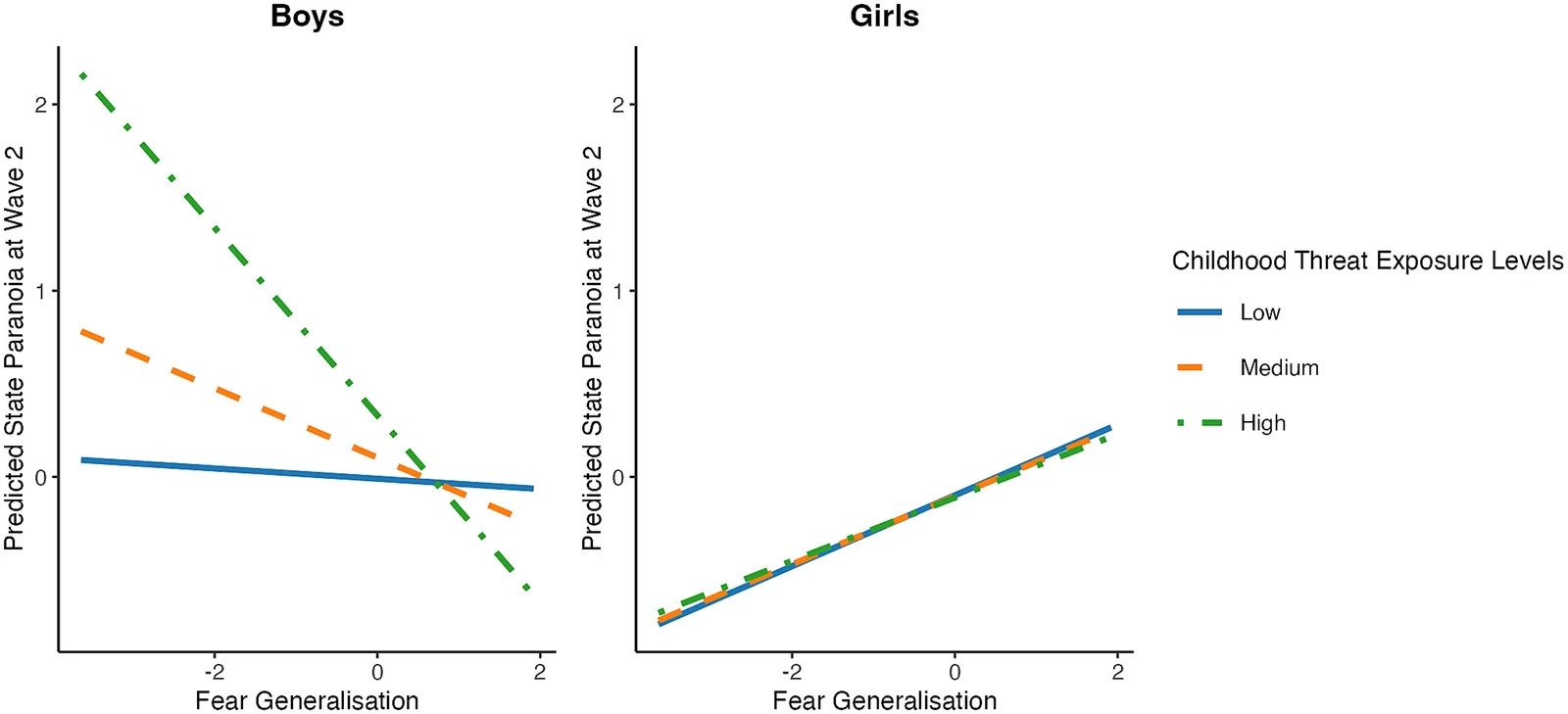
Gender Differences in the Interaction between Fear Generalization and Childhood Threat Exposure on Wave 2 State Paranoia. *Note.* CTE was treated as a continuous variable. Lines depict predicted values at the 25th (low), 50th (medium), and 75th (high) percentiles for visualization of the interaction. Abbreviation: CTE = childhood threat exposure.

## Examining Changes in State Paranoia Symptoms Over Time

### Threat–Safety Discrimination

In the model including only main effects, state paranoia at wave 1 significantly predicted state paranoia at wave 2 (β = 0.40, SE = 0.10, 95% CI [0.19, 0.60], *P* < .001), suggesting substantial temporal stability in state paranoia levels across the 18-month period.

The 2-way interaction between wave 1 TSD and wave 1 CTE did not reach significance in the second model (β = 0.14, SE = 0.09, 95% CI [−0.04, 0.32], *P* = .129). Also, wave 1 TSD was not significantly associated with changes in wave 2 state paranoia over time (β = 0.08, SE = 0.10, 95% CI [−0.12, 0.29], *P* = .406). Neither gender nor age predicted changes in state paranoia.

The TSD × CTE interaction explained an additional proportion of variance corresponding to a small incremental effect (Cohen’s *f*^2^ = .053). The interaction estimate was accompanied by a relatively wide confidence interval (β = 0.21, 95% CI [0.02, 0.40], *P* = .035), indicating uncertainty regarding the magnitude and precision of the effect.

### Fear Generalization

As expected, state paranoia at wave 1 also significantly predicted state paranoia at wave 2 in the model including FG (β = 0.41, SE = 0.10, 95% CI [0.21, 0.61], *P* < .001).

However, FG at wave 1 was not significantly associated with changes in wave 2 state paranoia (β = −0.05, SE = 0.10, 95% CI [−0.25, 0.16], *P* = .651). Similarly, gender and age were not significant predictors.

Second, the 2-way interaction between wave 1 FG and wave 1 CTE was not statistically significant (β = −0.09, SE = 0.12, 95% CI [−0.31, 0.14], *P* = .462).

Additionally, in the final model, the 3-way interaction among wave 1 FG, wave 1 CTE, and gender did remain significant (β = 0.51, SE = 0.26, 95% CI [0.00, 1.02], *P* = .048), confirming the gender-specific moderation of this relationship.

## Discussion

### Findings

This study aimed to investigate whether early adversity-related alterations in fear learning processes, TSD and FG, prospectively predict state paranoia during adolescence.

Contrary to our a priori hypotheses, the findings did not support the proposed prospective role of fear-learning processes in adolescent state paranoia. First, neither TSD nor FG at wave 1 directly predicted state paranoia at wave 2, indicating that poorer discrimination and greater generalization were not associated with higher subsequent paranoia at the group level. Second, although CTE moderated the association between TSD and later paranoia, the observed effect was opposite to our prediction: among adolescents with higher CTE, better rather than poorer TSD was associated with higher state paranoia. In contrast, the hypothesized 2-way interaction between FG and CTE was not significant. Third, the expected gender-specific effect was not observed. Rather than greater FG predicting higher paranoia in girls, a significant FG × CTE × gender interaction indicated that, among boys with higher CTE, lower rather than greater FG was associated with higher subsequent paranoia, whereas no significant association was found in girls. Finally, neither TSD nor FG predicted changes in state paranoia over the 18-month follow-up after controlling for baseline paranoia. Although the FG × CTE × gender interaction remained statistically significant in these analyses, it was again opposite to the hypothesized direction.

Taken together, none of the 4 primary hypotheses were fully supported. The observed interaction effects therefore generate alternative hypotheses regarding the relationship between CTE, fear-learning processes, and later paranoia, but because they emerged in the absence of significant group-level associations and were partly opposite to the predicted directions, they should be interpreted as exploratory findings requiring replication rather than as confirmation of the proposed theoretical framework.

### Threat–Safety Discrimination and State Paranoia

Baseline TSD was not directly associated with later state paranoia at the group level. However, an unexpected interaction emerged whereby, among adolescents with higher levels of CTE, better rather than poorer TSD was associated with higher state paranoia at follow-up. Because this interaction was opposite to our original hypothesis, it should be interpreted cautiously as an exploratory finding rather than as confirmation of the proposed theoretical framework.

One tentative interpretation of this unexpected pattern is provided by environmental calibration and latent vulnerability accounts, which propose that individuals growing up in threatening environments may develop adaptations that prioritize the detection and differentiation of potential danger. From this perspective, more precise TSD could reflect a more narrowly tuned threat-processing system in adolescents exposed to higher levels of childhood threat.^5^^,^^41^^,^^42^ This tentative interpretation is broadly consistent with evidence that childhood maltreatment is associated with altered amygdala responsivity to social threat.^43^ Within such contexts, more precise discrimination of threatening cues could be advantageous for detecting potential danger. However, it is also possible that, when these adolescents later encounter relatively safe yet socially ambiguous situations, such as those simulated in the present VR environment, this heightened sensitivity to potential threat could contribute to more suspicious or malevolent interpretations of others’ intentions, thereby increasing state paranoia. Nevertheless, the present findings do not directly test this proposed mechanism, and alternative explanations cannot be ruled out. The observed pattern should therefore be considered preliminary and requires replication before being interpreted as evidence for a “better safe than sorry” strategy. The observed sex differences, with effects specific to boys, are consistent with prior EMBRACE findings showing differential FG patterns between boys and girls.^5^ Although we did not observe a significant effect in girls, the broader literature suggests that girls may be more vulnerable to internalizing disorders following CA.^1^^,^^44^

### Fear Generalization, Gender, and State Paranoia

Contrary to our hypotheses, the effects of fear learning on later state paranoia did not follow the expected pattern. In particular, the effect of FG emerged in the opposite direction and was primarily observed in boys.

Baseline FG did not predict later state paranoia at the group level, nor was the FG × CTE interaction significant. Thus, the hypothesized association between greater FG and higher state paranoia was not supported. However, a FG × CTE × gender interaction emerged and replicated across analyses, but in a direction opposite to our predictions. Specifically, in males, the association between FG and state paranoia depended on CTE: under high CTE, *lower* FG was associated with *higher* state paranoia, whereas at low or moderate levels of exposure, FG showed little association with state paranoia. In females, although higher FG consistently predicted higher state paranoia irrespective of CTE level, the results were not statistically significant in the present sample. These findings suggest that FG may operate differently in relation to state paranoia than initially hypothesized, particularly in threat-exposed boys, pointing to distinct gender-specific mechanisms rather than a uniform risk effect.

Although these interaction effects should be interpreted as exploratory, they may generate alternative hypotheses regarding pathways through which CTE could influence threat-processing systems and later paranoid ideation.^45^ Consistent with this tentative interpretation, in boys exposed to high levels of childhood threat, lower FG, rather than heightened generalization as typically observed in anxiety, was associated with elevated state paranoia.^1^^,^^10^^,^^13^^,^^46^ One possible explanation is that state paranoia may develop through a cognitive-social pathway, characterized by conservative interpretations of ambiguous social cues, whereas anxiety and depression may reflect heightened affective reactivity and emotional generalization.^18^^,^^20^^,^^26^ Alternatively, reduced FG may reflect a more narrowly tuned threat-learning system that, in the context of early adversity, promotes conservative interpretations of ambiguous cues. If supported by future studies, this pattern could be compatible with a “better safe than sorry” strategy that prioritizes the detection of potential threat under conditions of uncertainty. Such an interpretation is broadly consistent with models linking fear learning and TSD to avoidance and defensive responding,^47^^,^^48^ and with the possibility that paranoia may involve a distinct cognitive-appraisal pathway rather than the affective overgeneralization typically implicated in anxiety disorders.^49^ Nevertheless, this proposed mechanism remains speculative and requires direct empirical testing.

### Course of Paranoia Throughout Adolescence

Another key finding was wave 1 state paranoia robustly predicting wave 2 state paranoia across 18 months. This finding is consistent with longitudinal research indicating that adolescent paranoia and mistrust may persist over time and are influenced by broader psychopathology, particularly internalizing symptoms.^50^^,^^51^ The absence of significant direct main effects of TSD and FG on later state paranoia is also broadly consistent with cognitive accounts that emphasize higher-order interpretation and attribution processes in the maintenance of paranoid ideation.^52–54^ Within these multifactorial models, fear-learning mechanisms may represent one of several potential vulnerability mechanisms; however, the present findings do not demonstrate that they independently contribute to the persistence of paranoia. Instead, the development and maintenance of paranoid ideation in interpersonal contexts may depend more strongly on social-cognitive processes, such as negative interpretation biases and maladaptive attributional styles.

### Strengths and Limitations

This study presents several notable strengths. First, the longitudinal and prospective design, spanning 2 assessment waves approximately 18 months apart, enables the examination of temporal ordering and prospective associations between early fear learning processes (TSD and FG) and later paranoia, addressing a major limitation of previous cross-sectional studies. Second, the multi-method approach, combining classical conditioning paradigms with immersive VR environments, provides both experimental control and ecological validity. The VR-based social settings (school canteen and bar) offered realistic simulations of social interactions, enhancing the authenticity of the paranoia assessments beyond what is achievable with self-report methods. Third, incorporating CTE as a moderator allowed the study to capture individual differences in developmental trajectories of paranoia, aligning with dimensional models of adversity that distinguish between threat and deprivation pathways. Moreover, the inclusion of gender as a moderating variable highlights important sex-specific mechanisms in FG and paranoia formation, an area often overlooked in previous conditioning research. Lastly, by focusing on paranoia rather than broader internalizing symptoms, the study extends fear-learning research beyond anxiety-focused frameworks and highlights the importance of distinguishing between affective and cognitive–appraisal pathways in developmental psychopathology.

Despite these strengths, several limitations should be acknowledged. The exclusion of psychophysiological data, due to technical issues, restricted the ability to integrate physiological measures with behavioral indices of fear learning, reducing insight into underlying neural and emotional processes. Additionally, slight inconsistencies in measurement across waves, specifically, the use of a 5-item SSPS at wave 1 and a 10-item version at wave 2, may affect direct comparability of paranoia scores, despite good internal consistency. Furthermore, the wave 1 and wave 2 assessments differed not only in the number of SSPS items but also in the VR social environments and the developmental stage of the participants. Although mean item scores were used to improve comparability, this approach does not establish measurement equivalence across waves. Consequently, the observed longitudinal association may reflect not only continuity in paranoid ideation but also contextual differences between the 2 VR paradigms, differences in scale structure, and developmental changes in the interpretation of socially ambiguous situations. Moreover, while the VR environments were adapted to the Belgian context, cultural specificity may limit generalizability to other adolescent populations where interpretations of social threat differ. Lastly, although the VR tasks were ecologically valid, the classical conditioning paradigm remains a simplified representation of real-world fear learning, using geometric stimuli that cannot fully capture the complexity of social threat cues implicated in paranoia. The analyses also did not adjust for several variables that may be relevant to both fear-learning processes and paranoia, including anxiety, depressive symptoms, trait paranoia, broader internalizing psychopathology, and cognitive functioning. This limits our ability to determine whether the observed associations are specific to state paranoia or instead reflect broader emotional or cognitive vulnerability. This concern is particularly relevant to FG, given its established association with anxiety-related processes. Accordingly, the present findings should not be interpreted as demonstrating mechanisms specific to paranoia.

The statistical robustness of the moderation findings should also be considered. Post hoc power analyses indicated limited power to detect both the TSD × CTE interaction (approximately 59%) and the FG × CTE × gender interaction (approximately 57%). Both effects were small in incremental magnitude and were accompanied by relatively wide confidence intervals. Furthermore, several interaction models were examined without formal adjustment for multiple comparisons. These findings should therefore be interpreted cautiously and require replication in larger, adequately powered independent samples.

## Conclusion and Future Directions

This study provides prospective evidence that fear-learning processes may be relevant to adolescent paranoia in the context of CTE, although the observed associations differed from our original hypotheses. While TSD and fear generalization did not predict later paranoia at the group level, better TSD and, in boys, reduced fear generalization were associated with elevated state paranoia among adolescents exposed to higher levels of childhood threat in immersive VR social environments. These findings should be interpreted cautiously because they emerged from interaction effects that were partly opposite to the predicted directions. Nevertheless, they raise the possibility that CTE may shape how fear-learning processes relate to paranoid interpretations in socially ambiguous contexts. More tentatively, these findings may indicate that early adversity shapes threat-processing systems toward heightened vigilance and more conservative interpretations of ambiguity. From this perspective, paranoid ideation may reflect a tentative “better safe than sorry” response to unpredictable environments, prioritizing the detection of potential threat while potentially increasing mistrust in relatively safe social contexts. This interpretation is consistent with the observed pattern but remains preliminary and requires direct testing and independent replication.

Future research should directly test the proposed mechanisms by integrating fear-learning paradigms with psychophysiological and neurobiological measures and by examining social–cognitive processes such as interpretation bias and intolerance of uncertainty as mediators.^55^ Methodologically, future longitudinal studies should use adequately powered designs, and employ equivalent or systematically comparable VR paradigms and paranoia measures across assessment waves. Including theoretically relevant variables, such as anxiety, depressive symptoms, trait paranoia, broader internalizing psychopathology, and cognitive functioning, will also be important for determining whether the observed associations are specific to paranoia or reflect broader emotional vulnerability. Replication in larger, independent, and more diverse samples is needed to clarify potential sex-specific pathways. Longitudinal studies spanning childhood to late adolescence will be essential for determining the stability and malleability of these threat-processing patterns and their implications for prevention and intervention in threat-exposed youth.

## Supplementary Material

Supplementary_Table_S1_VR_Items_sgag036

## Data Availability

The data underlying the findings of this study are available from the corresponding author upon reasonable request.

## References

[1] McLaughlin KA, Sheridan MA, Lambert HK. Childhood adversity and neural development: deprivation and threat as distinct dimensions of early experience. *Neurosci Biobehav Rev*. 2014;47:578-591. 10.1016/j.neubiorev.2014.10.012 PMC4308474 25454359

[2] Sheridan MA, McLaughlin KA. Dimensions of early experience and neural development: deprivation and threat. *Trends Cogn Sci*. 2014;18:580. 10.1016/j.tics.2014.09.001 PMC4252647 25305194

[3] Teicher MH, Gordon JB, Nemeroff CB. Recognizing the importance of childhood maltreatment as a critical factor in psychiatric diagnoses, treatment, research, prevention, and education. *Mol Psychiatry*. 2022;27:1331-1338. 10.1038/s41380-021-01367-9 PMC8567985 34737457

[4] McLaughlin KA, Weissman DG, Bitrán D. Childhood adversity and neural development: a systematic review. *Annu Rev Dev Psychol*. 2019;1:277-312. 10.1146/annurev-devpsych-121318-084950 PMC7243625 32455344

[5] Samaey C, Lecei A, Jackers M, et al. Childhood adversity is associated with reduced threat-safety discrimination and increased fear generalization in 12- to 16-year-olds. *J Child Psychol Psychiatry*. 2025;66:821-833. 10.1111/jcpp.14092 39670330

[6] Qiao Z, van der Donck S, Mazereel V, et al. Implicit neural sensitivity for negatively valued social and non-social visual scenes in young adults exposed to childhood adversity. *Psychol Med*. 2025;55:e37. 10.1017/S0033291725000029 PMC12017353 39930797

[7] Qiao Z, Samaey C, Jennen L, et al. Reduced threat-safety discrimination and generally enhanced generalization responses in adversity-exposed youth with emerging psychiatric symptoms. *BMC Psychiatry*. 2025;25:797. 10.1186/s12888-025-07254-9 PMC12362923 40830778

[8] Klingelhöfer-Jens M, Hutterer K, Schiele MA, et al. Reduced discrimination between signals of danger and safety but not overgeneralization is linked to exposure to childhood adversity in healthy adults. *eLife*. 2025;12:RP91425. 10.7554/eLife.91425 PMC11841987 39976327

[9] Lecei A, van Winkel R. Hippocampal pattern separation of emotional information determining risk or resilience in individuals exposed to childhood trauma: linking exposure to neurodevelopmental alterations and threat anticipation. *Neurosci Biobehav Rev*. 2020;108:160-170. 10.1016/j.neubiorev.2019.11.010 31743725

[10] Lissek S, Kaczkurkin AN, Rabin S, et al. Generalized anxiety disorder is associated with overgeneralization of classically conditioned fear. *Biol Psychiatry*. 2014;75:909-915. 10.1016/j.biopsych.2013.07.025 PMC3938992 24001473

[11] Lissek S, Biggs AL, Rabin SJ, et al. Generalization of conditioned fear-potentiated startle in humans: experimental validation and clinical relevance. *Behav Res Ther*. 2008;46:678-687. 10.1016/j.brat.2008.02.005 PMC2435484 18394587

[12] Cooper SE, van Dis EAM, Hagenaars MA, et al. Conditioned fear generalization in anxiety-related disorders: a meta-analysis. *Neuropsychopharmacology*. 2022;47:1652-1661. 10.1038/s41386-022-01332-2 PMC9283469 35501429

[13] Michalopoulou PG, Surguladze S, Morley LA, et al. Facial fear processing and psychotic symptoms in schizophrenia: functional magnetic resonance imaging study. *Br J Psychiatry*. 2008;192:191-196. 10.1192/bjp.bp.106.032649 18310578

[14] Smith KE, Pollak SD. Early life stress and development: potential mechanisms for adverse outcomes. *J Neurodev Disord*. 2020;12:34. 10.1186/s11689-020-09337-y PMC7745388 33327939

[15] Hu L, Stamoulis C. Strength and resilience of developing brain circuits predict adolescent emotional and stress responses during the COVID-19 pandemic. *Cereb Cortex*. 2024;34:bhae164. 10.1093/cercor/bhae164 PMC11484496 38669008

[16] Freeman D, Garety PA, Kuipers E, Fowler D, Bebbington PE. A cognitive model of persecutory delusions. *Br J Clin Psychol*. 2002;41:331-347. 10.1348/014466502760387461 12437789

[17] Green MJ, Phillips ML. Social threat perception and the evolution of paranoia. *Neurosci Biobehav Rev*. 2004;28:333-342. 10.1016/j.neubiorev.2004.03.006 15225975

[18] Freeman D, Garety P. Advances in understanding and treating persecutory delusions: a review. *Soc Psychiatry Psychiatr Epidemiol*. 2014;49:1179-1189. 10.1007/s00127-014-0928-7 PMC4108844 25005465

[19] Ellis BJ, Sheridan MA, Belsky J, McLaughlin KA. Why and how does early adversity influence development? Toward an integrated model of dimensions of environmental experience. *Dev Psychopathol*. 2022;34:447-471. 10.1017/S0954579421001838 35285791

[20] Bentall RP, Corcoran R, Howard R, Blackwood N, Kinderman P. Persecutory delusions: a review and theoretical integration. *Clin Psychol Rev*. 2001;21:1143-1192. 10.1016/S0272-7358(01)00106-4 11702511

[21] Bentall RP, de Sousa P, Varese F, et al. From adversity to psychosis: pathways and mechanisms from specific adversities to specific symptoms. *Soc Psychiatry Psychiatr Epidemiol*. 2014;49:1011-1022. 10.1007/s00127-014-0914-0 24919446

[22] Dunsmoor JE, Paz R. Fear generalization and anxiety: behavioral and neural mechanisms. *Biol Psychiatry*. 2015;78:336-343. 10.1016/j.biopsych.2015.04.010 25981173

[23] Dunsmoor JE, Prince SE, Murty VP, Kragel PA, LaBar KS. Neurobehavioral mechanisms of human fear generalization. *Neuroimage*. 2011;55:1878-1888. 10.1016/j.neuroimage.2011.01.041 PMC3062636 21256233

[24] Morriss J, Macdonald B, van Reekum CM. What is going on around here? Intolerance of uncertainty predicts threat generalization. *PLoS One*. 2016;11:e0154494. 10.1371/journal.pone.0154494 PMC4864232 27167217

[25] Greenburgh A, Bell V, Raihani N. The roles of coalitional threat and safety in paranoia: a network approach. *Br J Clin Psychol*. 2022;61:541-555. 10.1111/bjc.12342 34724219

[26] Inan C, Samaey C, Vansteelandt K, et al. A prospective study of VR-assessed state paranoia and anxiety suggests threat-related developmental pathways in adolescence. *Soc Psychiatry Psychiatr Epidemiol*. 2026;61:1617‑1627. 10.1007/s00127-026-03061-z 41824017

[27] Gayer-Anderson C, Knowles G, Beards S, et al. Immersive virtual reality as a novel approach to investigate the association between adverse events and adolescent paranoid ideation. *Soc Psychiatry Psychiatr Epidemiol*. 2024;60:305‑318. 10.1007/s00127-024-02701-6 PMC11839835 38942902

[28] Riches S, Pisani S, Bird L, Rus-Calafell M, Garety P, Valmaggia L. Virtual reality-based assessment and treatment of social functioning impairments in psychosis: a systematic review. *Int Rev Psychiatry*. 2021;33:337-362. 10.1080/09540261.2021.1918648 34121587

[29] Freeman D, Pugh K, Green C, Valmaggia L, Dunn G, Garety P. A measure of state persecutory ideation for experimental studies. *J Nerv Ment Dis*. 2007;195:781. 10.1097/NMD.0b013e318145a0a9 17984781

[30] Freeman D, Pugh K, Antley A, et al. Virtual reality study of paranoid thinking in the general population. *Br J Psychiatry*. 2008;192:258-263. 10.1192/bjp.bp.107.044677 18378984

[31] Freeman D, Reeve S, Robinson A, et al. Virtual reality in the assessment, understanding, and treatment of mental health disorders. *Psychol Med*. 2017;47:2393-2400. 10.1017/S003329171700040X PMC5964457 28325167

[32] Veling W, Counotte J, Pot-Kolder R, van Os J, van der Gaag M. Childhood trauma, psychosis liability and social stress reactivity: a virtual reality study. *Psychol Med*. 2016;46:3339-3348. 10.1017/S0033291716002208 27619196

[33] Lenaert B, van de Ven V, Kaas AL, Vlaeyen JWS. Generalization on the basis of prior experience is predicted by individual differences in working memory. *Behav Ther*. 2016;47:130-140. 10.1016/j.beth.2015.10.001 26763503

[34] Peirce JW . PsychoPy—psychophysics software in Python. *J Neurosci Methods*. 2007;162:8-13. 10.1016/j.jneumeth.2006.11.017 PMC2018741 17254636

[35] Riches S, Elghany S, Garety P, Rus-Calafell M, Valmaggia L. Factors affecting sense of presence in a virtual reality social environment: a qualitative study. *Cyberpsychol Behav Soc Netw*. 2019;22:288-292. 10.1089/cyber.2018.0128 30802148

[36] Bradley MM, Lang PJ. Measuring emotion: the self-assessment manikin and the semantic differential. *J Behav Ther Exp Psychiatry*. 1994;25:49-59. 10.1016/0005-7916(94)90063-9 7962581

[37] Valmaggia LR, Day F, Rus-Calafell M. Using virtual reality to investigate psychological processes and mechanisms associated with the onset and maintenance of psychosis: a systematic review. *Soc Psychiatry Psychiatr Epidemiol*. 2016;51:921-936. 10.1007/s00127-016-1245-0 27262562

[38] Kaczkurkin AN, Lissek S. Generalization of conditioned fear and obsessive-compulsive traits. *J Psychol Psychother*. 2013;3:7. 10.4172/2161-0487.1000137 PMC3932061 24567864

[39] Lissek S, Rabin S, Heller RE, et al. Overgeneralization of conditioned fear as a pathogenic marker of panic disorder. *Am J Psychiatry*. 2010;167:47-55. 10.1176/appi.ajp.2009.09030410 PMC2806514 19917595

[40] Finkelhor D, Hamby SL, Ormrod R, Turner H. The juvenile victimization questionnaire: reliability, validity, and national norms. *Child Abuse Negl*. 2005;29:383-412. 10.1016/j.chiabu.2004.11.001 15917079

[41] Samaey C, van der Donck S, Lecei A, et al. Between faces: childhood adversity is associated with reduced threat-safety discrimination during facial expression processing in adolescence. *BMC Med*. 2024;22:382. 10.1186/s12916-024-03610-w PMC11389258 39256825

[42] McCrory E, Ogle JR, Gerin MI, Viding E. Neurocognitive adaptation and mental health vulnerability following maltreatment: the role of social functioning. *Child Maltreat*. 2019;24:435-451. 10.1177/1077559519830524 30897955

[43] Hein TC, Monk CS. Neural response to threat in children, adolescents, and adults after child maltreatment: a quantitative meta-analysis. *J Child Psychol Psychiatry*. 2017;58:222-230. 10.1111/jcpp.12651 27778344

[44] Øien RA, Cogo-Moreira H, Nordahl-Hansen A, et al. Sex and age as moderators in the expression of internalizing and externalizing behaviors: insights from the child behavior checklist. *BMC Psychol*. 2025;13:1211. 10.1186/s40359-025-03529-8 PMC12581308 41184907

[45] Sadikova E, Weissman DG, Rosen ML, et al. Identifying cognitive, affective, and developmental mechanisms linking threat and deprivation with adolescent psychopathology. *J Child Psychol Psychiatry*. 2025;66:612-623. 10.1111/jcpp.14067 PMC12018145 39478358

[46] Kausche FM, Carsten HP, Sobania KM, Riesel A. Fear and safety learning in anxiety- and stress-related disorders: an updated meta-analysis. *Neurosci Biobehav Rev*. 2025;169:105983. 10.1016/j.neubiorev.2024.105983 39706234

[47] Krypotos AM, Effting M, Kindt M, Beckers T. Avoidance learning: a review of theoretical models and recent developments. *Front Behav Neurosci*. 2015;9:189. 10.3389/fnbeh.2015.00189 PMC4508580 26257618

[48] Dymond S, Dunsmoor JE, Vervliet B, Roche B, Hermans D. Fear generalization in humans: systematic review and implications for anxiety disorder research. *Behav Ther*. 2015;46:561-582. 10.1016/j.beth.2014.10.001 26459838

[49] Grupe DW, Nitschke JB. Uncertainty and anticipation in anxiety: an integrated neurobiological and psychological perspective. *Nat Rev Neurosci*. 2013;14:488-501. 10.1038/nrn3524 PMC4276319 23783199

[50] Catone G, Senese VP, Pascotto A, Pisano S, Broome MR. The developmental course of adolescent paranoia: a longitudinal analysis of the interacting role of mistrust and general psychopathology. *Eur Child Adolesc Psychiatry*. 2025;34:1415-1424. 10.1007/s00787-024-02563-y PMC12000268 39207495

[51] Bird JC, Waite F, Rowsell E, Fergusson EC, Freeman D. Cognitive, affective, and social factors maintaining paranoia in adolescents with mental health problems: a longitudinal study. *Psychiatry Res*. 2017;257:34-39. 10.1016/j.psychres.2017.07.023 28715666

[52] Freeman D . Persecutory delusions: a cognitive perspective on understanding and treatment. *Lancet Psychiatry*. 2016;3:685-692. 10.1016/S2215-0366(16)00066-3 27371990

[53] Trotta A, Kang J, Stahl D, Yiend J. Interpretation bias in paranoia: a systematic review and meta-analysis. *Clin Psychol Sci*. 2021;9:3-23. 10.1177/2167702620951552

[54] Freeman D, Loe BS. Explaining paranoia: cognitive and social processes in the occurrence of extreme mistrust. *BMJ Ment Health*. 2023;26:e300880. 10.1136/bmjment-2023-300880 PMC10649488 37945313

[55] Carpentier N, Hermans D, Scheveneels S. Predicting emotional distress based on acquisition, extinction, avoidance, and generalization learning. *Depress Anxiety*. 2024;41:6366269. 10.1155/2024/6366269 PMC11918936 40226683

