# Supplementary material for: Better Safe Than Sorry? Exploratory Associations Between Fear-Learning Processes and Later Paranoia in Threat-Exposed Adolescents in Standardized Virtual Reality Environments
Source: Schizophr Bull Open. 2026 Sep 12;7(1):sgag036. doi: 10.1093/schizbullopen/sgag036 (PMC13619336; doi:10.1093/schizbullopen/sgag036)
Supplement: Supplementary file 1 [file supplementary_table_s1_vr_items_sgag036.docx]

**Supplementary Table S1**
**State Social Paranoia Scale (SSPS) Items Presented in the Two VR Waves**

| **Wave 1 Items** | **Wave 2 Items** |
| --- | --- |
| Someone was aggressive towards me | Someone was hostile towards me |
| Someone was trying to bully me | Someone had bad intentions towards me |
| Someone was trying to frighten me | Someone was trying to make me distressed |
| Someone had it in for me | Someone stared at me in order to upset me |
| Someone stared at me in order to upset me | Someone wanted me to feel threatened |
|  | Someone would have harmed me in some way if they could |
|  | Someone had it in for me |
|  | Someone was trying to intimidate me |
|  | Someone was trying to isolate me |
|  | Someone was trying to irritate me |
